# Providing a Nursing Care Plan as a Requirement for Secondary Prevention for People Living With HTLV-1

**DOI:** 10.3389/fmed.2022.854970

**Published:** 2022-04-25

**Authors:** Cintia Yolette Urbano Pauxis Aben-Athar, Edilson Coelho Sampaio, Denise Silva Pinto, Antonio Carlos Rosário Vallinoto, Izaura Maria Vieira Cayres Vallinoto

**Affiliations:** ^1^Faculdade de Enfermagem, Instituto de Ciências da Saúde, Universidade Federal do Pará, Belém, Brazil; ^2^Laboratório de Virologia, Instituto de Ciências Biológicas, Universidade Federal do Pará, Belém, Brazil; ^3^Programa de Pós-graduação em Biologia de Agentes Infecciosos e Parasitários, Instituto de Ciências Biológicas, Universidade Federal do Pará, Belém, Brazil; ^4^Laboratório de Estudos em Reabilitação Funcional, Núcleo de Medicina Tropical da Universidade Federal do Pará, Belém, Brazil

**Keywords:** HTLV-1, care plan, nursing, diagnostics, assistance

## Abstract

**Introduction:**

The absence of nursing care plans aimed at people living with HTLV-1 (PLHTLV) led us to develop and test a proposed nursing care plan based on the evaluation of 55 PLHTLV to outline interventions according to the clinical stage.

**Methods:**

After interviews with symptomatic patients, nursing diagnoses were made using the NANDA International Nursing Diagnoses (The International Nursing Knowledge Association). Subsequently, interventions were selected through the Classification of Nursing Interventions (NIC), and expected results were selected through the Classification of Nursing Outcomes (NOC).

**Results:**

The actual diagnoses included (ii) chronic pain, (iii) impaired urinary elimination, and (iv) sexual dysfunction; the health promotion diagnosis was (i) risk-prone health behavior; and the risk diagnoses were (i) risk of feeling powerless and (ii) risk of falls in adults. Nursing care must prevent the lack of adherence to monitoring, establish goals and promote family involvement. A safe home environment requires intervention for fall prevention. Full support in understanding pharmacological and non-pharmacological therapies for chronic pain is needed. Interventions allow patients with impaired urinary function to be reintroduced to society. For sexual dysfunction, it is necessary to discuss safe sex and behavioral changes. Regarding risk behaviors, it is necessary to guide the patient/family, adapt language to the education level of these individuals, and help them better accept the condition, among other guidelines.

**Conclusion:**

The development of a nursing care plan for PLHTLV is essential for preventing the rapid progression of disease and the improvement of the quality of life of PLHTLV and should be included in the multidisciplinary approach to the secondary level of prevention of HTLV-1.

## Introduction

Human T-lymphotropic virus 1 (HTLV-1) was the first human retrovirus to be isolated from lymphocytes from a person with T-cell lymphoma (1). Studies carried out in the Caribbean and Japan made it possible to detect anti-HTLV-1 antibodies in people who had a neurological picture of spastic paresis, with the lower limbs being the most affected (2–4). The first clinical findings showed that HTLV-1 was a neurotropic agent and that, somehow, it was associated with the pathogenesis of the disease (2), currently called HTLV-1-associated myelopathy (HAM) (5).

Even 40 years after its isolation, HTLV-1 infection continues to be neglected (6), with no efficient drug treatment (7, 8) that promotes healing. Currently, treatment has been administered in the clinical practice of many health professionals, with the application of palliative care associated with the use of some drugs aimed at improving the health status of people living with HTLV-1 (PLHTLV) and, consequently, their quality of life (9, 10).

In Brazil, the follow-up of less complex cases of HTLV-1 can be performed in primary health care (PHC), while more complex cases, in which there is a need for the diagnosis of diseases associated with infection or the elaboration of the clinical management of these patients, should be aimed at secondary or tertiary levels of health care (11). Assistance for PLHTLV takes place through multidisciplinary teams, and nursing deserves to be highlighted since, especially in Brazil, it is one of the professions with the highest number of professionals and the closest proximity to users of the Unified Health System (SUS). For this professional class, the nursing process represents a great achievement because it allows the organization, energization, improvement and individualization of the care process. In addition, it allows for humanized care that is complementary to the assistance of other health professionals (12).

However, in Brazil, there is a lack of information among health professionals (13), especially among nurses (14), which makes it difficult to assist and monitor PLHTLV, as nurses must be able to identify diagnoses and plan interventions that are appropriate to improve the quality of care provided to the patient (14, 15). Associated with this, HTLV infection remains a public health problem in Brazil, with the absence of effective public policies that allow the reduction of transmission and the use of therapeutic measures (11). In this sense, the importance of building effective care plans is highlighted based on validation studies of tests and the creation of protocols (11), which provide an improvement in quality of life (15) and reduce vulnerability among PLHTLV (13).

The care plan must provide parameters to monitor the clinical status of patients, assist in the education process regarding prevention measures, and encourage the promotion of care for both PLWHA and their families to improve quality of care and quality of life, reduce stigma and discrimination, direct care, and increase the visibility of the topic (13). Thus, the present study aimed to develop a nursing care plan based on the evaluation of 55 PLHTLV and to design interventions according to the clinical picture.

## Methods

### Study

This was an observational, descriptive and cross-sectional study. The research was carried out from August 2016 to December 2018 at the Laboratory of Clinical and Epidemiology of Endemic Diseases of the Nucleus of Tropical Medicine (NMT) and at the Laboratory of Studies in Functional Rehabilitation (LAERF) of the Federal University of Pará (UFPA).

### Sampling and Ethical Aspects

The present study was based on a spontaneous demand for care from patients diagnosed with HTLV who sought the service for periodic evaluation. Fifty-five PLHTLV were evaluated (34 asymptomatic and 21 symptomatic for HAM), over 18 years of age and who agreed to participate in the research, by signing the Free and Informed Consent Form (TLCE). The research was approved by the Research Ethics Committee of the Institute of Health Sciences (ICS) of the Federal University of Pará (UFPA) (CAAE: 55699316.6.0000.0018).

### Data Collection

As described previously (16), data collection took place through the clinical assessment of PLHTLV using a semistructured interview questionnaire, including the following questions: (i) identification; (ii) socio-economic data (marital status, profession/occupation, family income and education); (iii) epidemiological data (blood transfusion, injecting drug use, information on sexual relations, number of partners, use of contraceptive methods, history of STI, history of breastfeeding, family history of HTLV or HTLV-associated disease); and (iv) clinical data (time of HTLV infection, how the diagnosis was made, and questions about HAM symptoms). The following instruments were used: the SALSA Scale, a social participation scale, the Brazilian version of the SF-36 Quality of Life Questionnaire and the Summarized Pain Inventory.

After the evaluation of the patients, the following steps were performed: (a) identification of diagnoses; (b) formulation of nursing results; (c) initial proposal of nursing interventions; and (d) elaboration of a care plan.

After the patients were interviewed, the identification of nursing diagnoses was carried out through the use of the Nursing Diagnoses of NANDA International (The International Nursing Knowledge Association) definitions and classification 2021–2023 (17).

The Nursing Outcomes Classification (NOC) (18) was used to select the expected outcomes for each proposed intervention. Subsequently, the selection of nursing interventions was made using the Classification of Nursing Interventions (NIC) (19). The intervention consisted of a therapy that could be directed to both the patient and his or her family (19).

## Results

The evaluation of 55 PLHTLV allowed the construction of a nursing care plan to be used in clinical practice in an outpatient setting. This plan was organized according to specific nursing methodologies based on the NANDA-I taxonomy (2021–2023) (17).

The diagnoses are listed in Table 1 by domain to facilitate the identification of the main affected areas in the patients. Of the 13 domains established in the NANDA-I taxonomy (17), the patients in this research had seven affected areas and needed special attention from nurses during the consultation.

**Table 1:** NANDA-I nursing diagnoses of patients with HTLV-1.

**Nursing diagnosis**			
**Domains**	**Actual diagnoses**	**Related factors/associated conditions**	**Ultimate characteristics**
Activity/rest	Ineffective home maintenance behaviors	Insufficient family planning; Insufficient support system	• Impaired ability to maintain housing; • Difficulty maintaining a comfortable environment
Comfort	Chronic pain	Chronic musculoskeletal condition	• Change in ability to continue previous activities; • Self-report of pain intensity using a standardized pain scale; • Self-report of pain characteristics using a standardized pain instrument
Elimination and exchange	Impaired urinary elimination	Sensory-motor damage	• Urinary urgency
Sexuality	Sexual dysfunction	Change in bodily function	• Decreased sexual desire; • Unwanted change in sexual function; • Perceived sexual limitations
**Domains**	**Health promotion diagnosis**	**Related factors**	**Ultimate characteristics**
Health promotion	Risk-prone health behavior	Negative perception of recommended health care strategy	• Non-acceptance of the change in health status
**Domains**	**Risk diagnosis**	**Risk factors**	**Associated conditions**
Coping with or tolerating stress	Risk of feeling powerless	Progressive disease; Unpredictability of the course of the disease	• Insufficient knowledge to control the situation; • Aches; • Stigmatization
Security/protection	Risk of falls in adults	Impaired physical mobility; Chronic musculoskeletal pain; Decreased strength in the lower extremities	• Musculoskeletal diseases

The domains identified were (i) activity/rest, (ii) comfort, (iii) elimination and exchange, (iv) sexuality, (v) health promotion, (vi) coping/stress tolerance and (vii) safety/protection.

Thus, in Table 1, the following real nursing diagnoses are presented: (i) lack of adherence, (ii) impaired home maintenance, (iii) chronic pain, (iv) impaired urinary elimination and (v) sexual dysfunction. In addition, the health promotion diagnosis was risk-prone health behavior, and the following risk diagnoses were identified: risk of feeling helpless and risk of falls.

The identification of diagnoses allows the nurse to draw up a care plan that must be followed for the proposed interventions and, later, must be evaluated to see if the expected results were achieved.

Table 2 presents the care plan established for the assessment and monitoring of PLHTLV. Notably, such a plan should be used as a guide for nurses' conduct during care for PLHTLV since such diagnoses were common to the patients evaluated. It is relevant to highlight that each patient must be individually and critically evaluated so that the nurse can implement care directed to the real needs of PLHTLV.

**Table 2:** Nursing care plans for HTLV-1-infected patients.

**Nursing diagnoses (NANDA-I)**	**Nursing interventions (NIC)**	**Expected results (NOC)**
**(i) Impaired home maintenance:** Inability to independently maintain a safe environment to promote growth.	**1. Environment control - security:** • Identify safety hazards in the environment; • Remove hazards from the environment where possible; • Modify the environment to minimize hazards and risks; • Provide adaptation devices in order to increase the safety of the environment.	**1. Safe home environment:** The patient must take physical measures to minimize environmental factors capable of causing injury or physical injury at home. **INDICATORS:** • Provisions for internal lighting; • Placement of handrails; • Provision of auxiliary devices in an accessible location; • Organization of furniture in order to reduce risks.
**(ii) Chronic pain:** Unpleasant sensory and emotional experience associated with actual or potential tissue damage or described in terms of such damage (International Association for the Study of Pain); sudden or slow onset, mild to severe, constant or recurrent, with no anticipated or predictable end, lasting longer than 3 months.	**1. Pain control:** • Conduct a complete pain assessment, including location, characteristics, onset/duration, frequency, quality, intensity and severity, in addition to precipitating factors; • Determine the impact of pain on quality of life; • Investigate with the patient the factors that relieve/worsen pain; • Use a properly developed assessment method that allows the monitoring of changes in pain (e.g., journaling); • Teach the use of non-pharmacological techniques; • Investigate the patient's current use of pharmacological pain relief methods.	**1. Pain control:** The patient must report personal actions to control the pain. **INDICATORS:** • Using a diary to monitor symptoms over time; • Use of non-analgesic relief measures; • Reporting of uncontrolled changes in pain symptoms to the healthcare professional. **2. Customer satisfaction - Pain management:** The patient must achieve a positive perception of nursing care to relieve pain. **INDICATORS:** • Pain level monitored regularly; • Actions implemented to provide comfort; • Safety issues addressed regarding pain medication use.
**(iii) Impaired urinary elimination:** Dysfunction in the elimination of urine.	**1. Control of urinary elimination:** • Monitor urinary elimination, including frequency, consistency, odor, volume, and color, as appropriate; • Teach the patient the signs and symptoms of a urinary tract infection; • Assist the patient in developing a toileting routine, as appropriate. **2**. **Urinary habit training:** • Keep a specific continence record for 3 days to establish the urinary elimination pattern; • Establish the range of initial times to use the toilet, based on the urinary elimination pattern and the usual routine; • Discuss the daily continence record with staff to provide reinforcement and encourage adherence to toileting schedules; • Maintain toileting schedules to help establish and maintain a urinary elimination habit.	**1. Urinary elimination:** The patient should progress to urine storage and elimination. **INDICATORS:** • Elimination pattern; • Recognition of urgency.
**(iv) Sexual dysfunction**: The state in which the individual undergoes a change in sexual function, during the sexual response phases of desire, arousal, and/or orgasm that is seen as unsatisfactory, unrewarding, and inappropriate.	**1. Teaching safe sex:** • Reinforce condom use; • Educate the patient on the correct application and removal of condoms, as appropriate; • Discuss ways to convince the partner to use condoms; • Plan sex education classes for patient groups as appropriate. • **2**. **Behavior modification:** • Determine the patient's motivation to change; • Help the patient identify strengths and reinforce them; • Encourage the replacement of undesirable habits with desirable habits.	**1**. **Risk control: sexually transmitted infections (STIs):** The patient must present personal actions to prevent, eliminate or reduce behaviors associated with the sexually transmitted infection. **INDICATORS:** • Recognize the individual risks of STIs; • Recognize the personal consequences associated with STIs; • Commitment to exposure control strategies; • Warning to sexual partner(s) in the event of an STI.
**(v) Risk-Prone Health Behavior:** Impaired ability to modify lifestyle/behaviors in order to improve health status.	**1**. **Improvement in coping:** • Assess the patient's adaptation to changes in body image, if indicated; • Assess the impact of the patient's life situation on roles and relationships; • Use a calm approach; • Provide an atmosphere of acceptance; • Offer information about diagnosis, treatment and prognosis.	**1. Acceptance of the state of health:** The patient should report acceptance of a significant change in health status. **INDICATORS:** • Abandonment of the previous concept of personal health; • Recognition of the reality of the health situation; • Adaptation to changes in health status; • Performance of self-care tasks.
**(vi) Risk of feeling of powerlessness**: Risk of perceived lack of control over a situation and/or a person's ability to significantly affect an outcome.	**1. Learning facilitation:** • Start instruction only after the patient demonstrates readiness to learn; • Adapt instruction to the patient's level of knowledge and understanding; • Adapt the content to the patient's cognitive, psychomotor and/or affective abilities/difficulties; • Use familiar language; • Allow time for the patient to ask questions and discuss his or her concerns. • **2**. **Teaching about the disease process:** • Assess the patient's current level of knowledge; • Review what the patient knows about the condition; • Describe the common signs and symptoms of the disease, as appropriate; • Discuss lifestyle changes that may be necessary to avoid future complications and/or control the disease process; • Describe possible chronic complications, as appropriate.	**1. Knowledge of the disease process:** The patient should gain a broad understanding of a specific disease process and the prevention of complications. **INDICATORS:** • Strategies to minimize disease progression; • Precautions to prevent complications from the disease.
**(vii) Risk of falls in adults**: Adult susceptibility to experiencing a fall event that can compromise health.	**1**. **Environment control:** • Create a safe environment for the patient; • Identify patient safety needs based on level of physical and cognitive functioning and previous behavioral history; • Remove environmental hazards (e.g., carpet); • Offer adaptation devices. • Have escorts if the patient is elderly or in a critical health condition.	**1**. **Behavior for fall prevention:** The patient should report personal or family caregiver actions to minimize risk factors capable of precipitating falls in the personal environment. **INDICATORS:** • Placement of support handrails if necessary; • Use of rubber mats in the shower area; • Use of laced shoes that fit well; • Offering of mobility assistance; • Removal of loose rugs; • Elimination of clumps of objects, spilled liquids and floor shine. **2**. **Knowledge: fall prevention:** The patient should report the extent of understanding about the prevention of falls. **INDICATORS:** • Correct use of auxiliary devices; • Correct use of safety devices; • Correct use of support bars.

The nurse must, at each consultation, reassess the patient and identify the diagnoses to establish new behaviors to be followed based on the care plan produced in this research. The routine periodic consultations allow the clinical follow-up of the patient, targeted care aimed at improving the quality of life, and sex education with the purpose of interrupting the chain of transmission of the virus.

## Discussion

Nursing consultations for PLHTLV are intended to promote self-care actions, institute care for recovery, and provide guidance for adaptation to the consequences imposed by diseases associated with the virus (20). In this sense, the care plan deserves to be highlighted, as it can be used as a tool to promote holistic care (21), but it requires planning and must be created based on nursing diagnoses, as these allow the identification of what achievements are desired and what interventions are needed (22).

Nursing must act based on two main aspects: the establishment of mutual goals and the promotion of family involvement. It is entirely relevant that professionals be trained to promote the correct orientation to patients (23). Family members, in turn, should be encouraged so that a support network can be established (24). In this way, a favorable environment is created to promote adherence to rehabilitation, as well as to enable the improvement of trust and, consequently, the improvement of the nurse/patient interpersonal relationship (25). It becomes possible for the patient to redefine many issues, such as their plans and dreams, which are often left aside after the diagnosis (23).

The diagnosis of ineffective home maintenance behaviors refers to the impossibility of independently leaving the safe environment. Environmental control has been instituted as an intervention to achieve a safe home environment (17–19). It is common for patients with HTLV-1 to report falls and even fear of falls (26); therefore, some changes in the environment in which the patient lives are necessary.

Pain causes not only physical discomfort but also functional limitations and limitations in carrying out activities (24); in addition, pain treatment is often tiring and does not always achieve positive results (27). Therefore, the teaching of pain control and/or monitoring becomes indispensable. Thus, pharmacological and non-pharmacological therapies must be maintained so that pain is controlled (28), and nursing must remain attentive to eliminate patients' possible doubts and to help them understand the therapies chosen by health team.

Another nursing diagnosis verified in the patients in this study was impaired urinary elimination, so the interventions suggested in the care plan included elimination control and urinary habit training, which aim, above all, for efficient urinary elimination (17–19). The neurological sequelae are painful, and many patients develop urinary disorders, which often make it impossible to carry out their usual activities. In this sense, the instructions and the promotion of bladder reestablishment allow the patient's reintroduction to society, with the accomplishment of their tasks and with the strengthening of ties (29).

Disabilities that occur as a result of the neurodegenerative process often cause the patient to lose privacy, as he or she becomes dependent on other people to perform self-care activities such as going to the bathroom (23). Furthermore, the increase in urinary frequency causes not only emotional damage but also social damage due to inherent isolation (23). As a result, the quality of life is affected, and as there is no treatment that totally improves this urinary issue, rehabilitation must be offered (30).

Sexual issues must be addressed during patient education practices, as diagnosis with HTLV-1 causes changes in the sexual life of PLHTLV (29). The condition of the person living with the virus, increased urinary frequency and sexual dysfunction change the routine of couples and, as a consequence, change the ability to experience pleasure and sometimes cause the separation of couples who previously had sexual and affective relationships (20). The diagnosis of sexual dysfunction was evident in this research, and due to its consequences, the following intervention was established: teaching safe sex and behavior modification (17–19).

Teaching and counseling actions are very important health promotion activities and therefore require professionals to know the implications of HTLV-1 infection, as there is a lack of knowledge among health professionals (31). Counseling should include questions about sexuality and the use of prevention measures and should also address pregnancy, prenatal care, childbirth and the postpartum period, in particular issues related to avoiding breastfeeding and using formulas to replace breastmilk (29).

The health promotion and risk diagnoses identified from the assessment of PLHTLV demand the following interventions: improving coping, facilitating learning and/or teaching the disease process and controlling the environment (17, 19).

HTLV-1 is associated with a chronic disease that promotes mobility impairment, which becomes an important factor in the risk of falls. To prevent and/or reduce this risk, the patient should be instructed to use mobility aids. However, even with the risk of falling, patients prefer to derive support from objects rather than use an aid (24). The importance of guidance is evident so that such risk is avoided.

Guidance can also be provided to help the patient and/or family member better accept the health condition, preventing risk-prone health behavior. Understanding allows for better acceptance and encourages the continuity of participation and engagement in activities in society, thus enabling the acquisition of a new attitude toward the disease (24).

The risk of feeling helpless was evidenced among the patients in this research, and for this situation to be avoided, it is necessary for nurses to teach the health and disease process, always clarifying the doubts of the patient and family members. Such instruction must be performed orally and/or in writing, with the language always being adapted according to the level of education of patients and their families (32), as has already occurred in other care centers for PLHTLV (20).

Within this context of health promotion, it is essential to carry out activities through nursing, either based on the guidelines, the care taught or the possibility of identifying risks and the use of timely interventions. This enables the patient to provide care not only for the treatment of the consequences of the pathology but also for himself or herself as a human being, thus providing more comprehensive care (20, 23).

Nursing is a profession that stands out in the context of health care for PLHTLV, given the possibility for nurses to work in different care contexts, from primary to tertiary health care, and to always seek to develop promotion activities and the protection and recovery of health according to the guidelines of the SUS. The care activity of nurses takes place through the nursing process, which consists of a scientific work methodology that directs care based on clinical findings. Thus, it is understood that the elaboration of a care plan for PLHTLV constitutes an advance for care to become more directed to the clinical characteristics of this specific public. Additionally, it enables the redefinition of the nurse's role in this context.

## Conclusions

The clinical evaluation of the patients in this research allowed the identification of nursing diagnoses that enabled the construction of a care plan, which aims to improve care for PLHTLV. The findings of this research confirm the importance of nurses being able to assess and identify diagnoses to enable the creation of more comprehensive care. Finally, the development of a nursing care plan allowed the planning of feasible actions that aim to improve and complement the multidisciplinary care offered to PLHTLV and their families, seeking to increase the perseverance, comfort and quality of life of patients.

## Data Availability Statement

The raw data supporting the conclusions of this article will be made available by the authors, without undue reservation.

## Ethics Statement

The studies involving human participants were reviewed and approved by Human Research Ethics Committee of the Health Sciences Institute of the Federal University of Pará (CAAE: 55699316.6.0000.0018). The patients/participants provided their written informed consent to participate in this study.

## Author Contributions

CA-A, DP, and AV conceived and designed the study. CA-A, DP, and ES assisted in patient recruitment and health care. CA-A, AV, and ICV wrote the manuscript. All authors read and approved the final manuscript.

## Funding

This study was supported by the National Council for Scientific and Technological Development (CNPq; # 301869/2017-0; and 442522/2019-3), Pan-American Health Organization (#SCON2021-00310), and the Federal University of Pará (PAPQ-2022).

## Conflict of Interest

The authors declare that the research was conducted in the absence of any commercial or financial relationships that could be construed as a potential conflict of interest.

## Publisher's Note

All claims expressed in this article are solely those of the authors and do not necessarily represent those of their affiliated organizations, or those of the publisher, the editors and the reviewers. Any product that may be evaluated in this article, or claim that may be made by its manufacturer, is not guaranteed or endorsed by the publisher.
